# Improvement in hand hygiene compliance. The impact of a regional strategy

**DOI:** 10.1186/1753-6561-5-S6-O67

**Published:** 2011-06-29

**Authors:** P Rodriguez-Perez, CC Mireia, RR Cristina, PM Rosa, JM Ana Belén, NR Cristina, PH Alberto

**Affiliations:** 1 Medicina Preventiva , Hsopital General Universitario Gregorio Marańon, Spain; 2 Subdireccion General de Calidad, Consejería de Sanidad de la Comunidad de Madrid, Madrid, Spain

## Introduction / objectives

Health-care workers (HCWs) adherence to hand hygiene (HH) guidelines is usually low. According to WHO recommendations, the key strategy components to improve the compliance is evaluation and feed-back.In this study we monitored HH compliance of HCWs by direct observation in Madrid and to asses the impact of an interventional strategy.

## Methods

We observed 33 Madrid’s Public Hospitals (more than 40.000 Health care workers) before and after the implementation of a regional intervention following WHO multimodal HH improvement strategy. It included different interventions but was specially focused in an online education tool with a play learning methodology. Our study involved a wide range of HWCs: dentists, physiotherapist, nurses, physicians and students. We performed a 6-week observation period, March-April 2010 and the same period in 2011.

## Results

Around 10.000 opportunities for HH were observed in each period. Compliance in the first period was 30.9% (95% C.I.:30-31.7). The second period is currently taking place, but an interim analysis reveals a HH compliance of a 47%(95% C.I.:44,92-49,38).

In the 1^st^ period the lowest compliance is achieved at the 2nd moment (14.6%; before an aseptic task), followed by the 1st moment (25.1%; before patient contact). In the second period the use of alcohol based solutions have increased reaching a 77% of all HH performed. The technique has also improved.

## Conclusion

The result of the regional strategy shows an important improvement in HH compliance.

We believe this improvement is having a impact in reducing health care associated infections. Further studies are needed to assure sustainability in time and impact in infection rates.

## Disclosure of interest

None declared.

